# Perspectives of Parents and Health Care Workers on Early Infant Male Circumcision Conducted Using Devices: Qualitative Findings From Harare, Zimbabwe

**DOI:** 10.9745/GHSP-D-15-00200

**Published:** 2016-07-02

**Authors:** Webster Mavhu, Karin Hatzold, Getrude Ncube, Shamiso Fernando, Collin Mangenah, Kumbirai Chatora, Owen Mugurungi, Ismail Ticklay, Frances M Cowan

**Affiliations:** a Centre for Sexual Health and HIV/AIDS Research (CeSHHAR), Harare, Zimbabwe; b University College London, London, United Kingdom; c Population Services International, Harare, Zimbabwe; d Ministry of Health and Child Care, Harare, Zimbabwe; e University of Zimbabwe College of Health Sciences, Harare, Zimbabwe

## Abstract

**Background:**

The World Health Organization (WHO) and the United Nations Children’s Fund (UNICEF) recommend early infant male circumcision (EIMC) for prevention of HIV. Here, we present findings from a qualitative study in Zimbabwe that assessed parental and health care workers' perspectives of EIMC conducted using devices.

**Methods:**

This qualitative study was nested within a trial of EIMC devices. Between January and May 2013, we held 4 focus group discussions (FGDs) and 12 in-depth interviews with parents and 12 in-depth interviews with clinicians (7 trial clinicians and 5 non-trial clinicians). We also conducted 95 short telephone interviews with parents who had arranged to bring their sons for EIMC but then defaulted.

**Results:**

Parents who had adopted EIMC spoke of their initial anxieties about the procedure. Additionally, they commented on both the procedure and outcome. Parents who decided against EIMC cited fear of harm, specifically the infant's death, penile injury, and excessive pain. Misperceptions about male circumcision in general and EIMC specifically were a significant barrier to EIMC adoption and were prevalent among health care workers as well as parents. In particular, the findings suggest strong parental concerns about the fate of the discarded foreskin. Parents who chose EIMC for their newborn sons felt that the procedure was safe and expressed satisfaction with the outcome. For their part, health care workers largely thought that EIMC was safe and that the outcome was aesthetically pleasing. They also felt that it would be feasible to offer wide-scale EIMC for HIV prevention in the public sector; they recommended strategies to increase EIMC uptake, in addition to highlighting a few concerns.

**Conclusions:**

The qualitative study enables us to better understand parental and health care workers' perspectives of EIMC conducted using devices, especially their perspectives on EIMC safety, feasibility, acceptability, and barriers. These findings will be used to design demand-generation activities that support wider adoption of EIMC.

## Abstract (precis)

Parents who opted for early infant male circumcision (EIMC) and health care workers felt EIMC was a safe and acceptable procedure that would likely become more widely adopted over time. Barriers to EIMC uptake such as parental fears of harm and cultural beliefs are potentially surmountable with adequate education and support.

## INTRODUCTION

In Africa, 14 countries are currently accelerating roll-out of voluntary medical male circumcision (VMMC).^1^^–^^5^ Modeling studies conducted between 2009 and 2011 suggested that circumcising males ages 15 to 49 years to reach 80% coverage within 5 years in these countries, and maintaining this coverage thereafter, could avert 3.4 million new HIV infections within 15 years and yield treatment and care savings of US$16.5 billion.^2^^,^^3^ In order to ensure that the protective effect of male circumcision is sustained in the longer term, the World Health Organization (WHO), the Joint United Nations Programme on HIV/AIDS (UNAIDS), and the United Nations Children’s Fund (UNICEF) also recommend that early infant male circumcision (EIMC)—that is performed within the first 60 days of life—be implemented alongside VMMC.^6^^,^^7^ Presuming high rates of uptake of EIMC, it will then be possible to phase out the “catch-up” adult VMMC as circumcised infants come of age.

Although EIMC’s impact on the HIV epidemic will take some time to realize, infant circumcision is ultimately likely to be more effective at preventing HIV acquisition than adult male circumcision, as the procedure is carried out long before the individual becomes sexually active, avoiding the risk associated with sex during the healing period.^8^ Like VMMC, EIMC will protect against some sexually transmitted infections and genital cancers in addition to HIV.^9^^,^^10^

Furthermore, studies estimate that EIMC is likely to be a cost-saving HIV prevention intervention in the long term, and less costly than VMMC.^11^^,^^12^ Projections suggest that providing universal access to male circumcision, including EIMC, in conjunction with other effective HIV prevention interventions, will reduce the overall cost of HIV epidemics driven by heterosexual transmission.^12^ EIMC can be viewed as background population-level protection for future generations.^8^

EIMC can be viewed as background population-level protection for future generations.

Since 2009, Zimbabwe has provided circumcision to over 600,000 adult and adolescent men. The program aims to reach 1.3 million 15- to 29-year-olds by 2017.^13^ Zimbabwe intends to offer EIMC alongside VMMC. Since large-scale EIMC for HIV prevention, or indeed for any reason, has never been practiced in Zimbabwe or, more widely, in Southern Africa, there are concerns about its feasibility and acceptability. Clearly, the acceptability of infant male circumcision will have a bearing on uptake, roll-out, and resulting effectiveness in preventing HIV. In Zimbabwe, there are also concerns about the feasibility of rolling out EIMC for HIV prevention within the context of existing health services, many of which are already overburdened and understaffed. Here, we present findings from a qualitative study that was nested within a trial of EIMC devices to assess in depth parental and health care workers’ perspectives with a view to informing demand creation and roll-out.

## METHODS

### Study Design

This qualitative study sought to complement an earlier study that explored the hypothetical feasibility and acceptability of EIMC among health care workers, parents, and the wider family (described in detail elsewhere^14^^,^^15^). Findings from the earlier study informed the design of an individually randomized non-inferiority trial that assessed the feasibility, safety, acceptability, and cost of rolling out EIMC using devices (AccuCirc and Mogen clamp) in Zimbabwe.^16^^,^^17^ During the trial, between January and June 2013, parents of newborn boys at a Harare clinic were invited to participate in this study. Some 150 eligible male infants were enrolled in the trial (13% uptake) and were circumcised at 6 to 54 days old by a doctor (n = 100 AccuCirc; n = 50 Mogen clamp).^16^^,^^17^

In brief, the Mogen clamp is a reusable stainless steel device that requires a new, sterile scalpel blade for each infant male circumcision.^18^ Circumcision using the Mogen clamp can (occasionally) result in partial or total amputation of the glans penis or removal of too little foreskin (in which case the remaining foreskin remains vulnerable to infection with HIV).^19^^–^^22^ The Atraumatic Circumcision (AccuCirc) device is an instrument made largely of plastic. It has a shielding ring that protects the glans penis, preventing laceration or amputation.^23^ The qualitative study reported here assessed in depth the actual (as contrasted with hypothetical) feasibility and acceptability of EIMC conducted using the 2 devices. EIMC was not available at the clinic outside the context of this trial.

### Qualitative Study Sampling and Data Collection

Between January and May 2013, 2 teams of trained and experienced researchers (1 team of male interviewers; 1 team of female interviewers) held in-depth interviews and focus group discussions (FGDs) with parents. Budgetary and time constraints limited the numbers of interviews, discussions, and phone calls. In all, 24 participants took part in in-depth interviews and another 38 in FGDs. An additional 95 parents took part in short phone interviews. No one declined to take part in the qualitative study.

We conducted in-depth interviews and FGDs with parents who had adopted EIMC for HIV prevention—3 interviews and 1 FGD with a total of 10 mothers; 3 interviews and 1 FGD with a total of 9 fathers. We selected these participants randomly from a list of parents who had adopted EIMC. We also conducted interviews and FGDs with parents who had declined to circumcise their newborn sons—3 interviews and 1 FGD with a total of 10 mothers, and 3 interviews and 1 FGD with a total of 9 fathers. We selected these participants randomly from a list of couples who had not adopted EIMC and had not been shortlisted for short phone interviews (see the Figure for qualitative study sampling).

**FIGURE:**
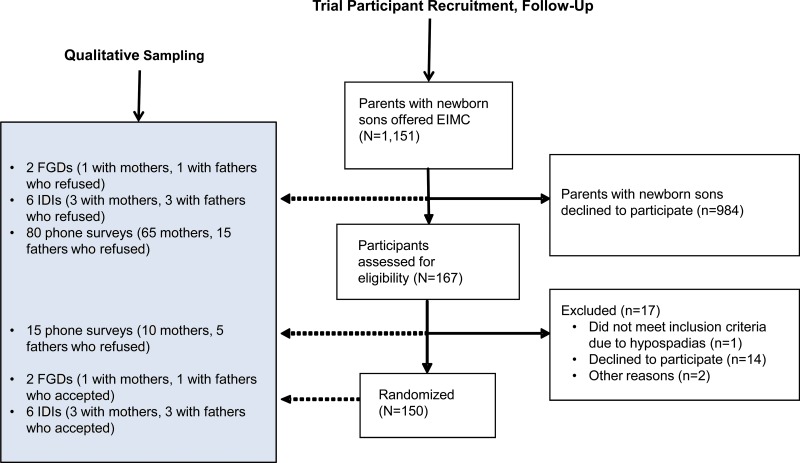
Sampling for the Qualitative Study Abbreviations: EIMC, early infant male circumcision; FGD, focus group discussion; IDI, in‐depth interview.

With parents who accepted EIMC, discussions explored, among other topics, perceptions of the procedure’s safety and final appearance, as well as whether they would recommend EIMC to other parents. With parents who declined EIMC, discussions explored reasons for not taking up the procedure, their fears and/or concerns, as well as what would need to change for them to opt for EIMC.

We also interviewed in depth the 4 doctors who performed EIMC during the comparative trial and the 3 EIMC study nurse-midwives who assisted doctors during the trial. We also interviewed in depth 5 female nurses who worked at the study clinic but were not directly involved in performing procedures or recruiting infants for the EIMC trial. The 5 nurses were purposively sampled to include a sister-in-charge, 2 nurse-midwives based in the maternity unit, and 2 registered general nurses based in the family health services clinic (the unit where babies and children are seen for immunizations and growth monitoring). Discussions with clinicians assessed actual acceptability of the procedure and obtained their views on the feasibility of offering wide-scale EIMC using either the AccuCirc device or Mogen clamp. Discussions also elicited clinicians’ perceptions of the safety of the procedure when conducted with either device.

Further, we conducted 95 short phone interviews with parents who had arranged to bring their sons for EIMC but then defaulted. The phone interviews sought to ascertain only the parents’ reasons for not bringing the infant for the procedure. In these phone interviews, we included 15 of the 17 parents (88%) who had gone through all study screening procedures (including providing locator information, comprehension of screening eligibility criteria, and responding to a one-time questionnaire) but did not eventually enroll. Of these 15, 10 were mothers and 5 were fathers. We selected the other 80 phone interview participants as follows: 65 women (10%) were randomly selected from a list of 650 mothers who had arranged to bring their sons for EIMC but then defaulted. An additional 15 men (10%) were randomly selected from a list of 150 fathers who had participated in weekend group meetings and had promised to bring their sons for EIMC but then defaulted (Figure). We contacted phone interview participants when their sons were no longer eligible for EIMC. We handwrote short statements of these phone conversations and later coded them.

FGDs lasted 2 to 2.5 hours; in-depth interviews lasted 45 minutes to 1 hour. All FGDs were conducted in Shona, the participants’ language. In-depth interviews were conducted in either English or Shona, depending on the participant’s preference. We audio-recorded all FGDs and in-depth interviews.

### Data Analysis

We transcribed the recorded interviews and discussions and, where necessary, translated them verbatim into English. We identified initial themes during the interviews and discussions; these themes informed an initial coding framework. Then, 3 researchers used this framework to code 5 in-depth interviews and all FGDs line by line on paper. This led us to add additional codes to the coding framework. We entered the transcripts into NVivo 10 (QSR International, Melbourne, Australia), a qualitative data storage and retrieval computer program. Trained and experienced researchers (authors CM and SF) coded each transcription separately, using the modified coding framework, taking note of any emerging new codes. If the coders disagreed over the interpretation of some codes, the senior social scientist (WM) met with the 2 researchers. The 3 would examine the codes and collectively agree on the standard forms to use for coding. We then revised the coding framework accordingly. WM checked concordance of the 2 researchers’ coding in addition to independently coding all transcripts. We typed the handwritten statements from the phone interviews and entered the information into a Microsoft Excel document. Researchers read the statements and assigned each statement a code based on its key words.

We grouped codes into categories and then identified emerging themes following the principles of thematic analysis.^24^^,^^25^ As we wrote up the findings, we illustrated themes and subthemes with verbatim quotations.

### Ethical Considerations

The study was approved by the Medical Research Council of Zimbabwe and the ethics committees of University College London and the London School of Hygiene and Tropical Medicine. We obtained written informed consent from participants on the day of the interview or discussion. At the start of each short phone interview, we sought verbal consent.

## RESULTS

Parents who had adopted EIMC spoke about their initial anxieties concerning the procedure. Additionally, they commented on both the procedure and its outcome. Parents who had decided against EIMC mentioned fear of harm, specifically the infant’s death, penile injury, and excessive pain. There were no discernible differences between mothers' and fathers' responses.

On the whole, health care workers thought EIMC was a safe procedure, and the outcome was aesthetically pleasing. Nearly all EIMC study doctors and nurses expressed preference for the AccuCirc device over the Mogen clamp. We detail these findings below.

### Parents Reported Initial Anxieties

Discussions suggested that most parents who had adopted EIMC initially had some anxieties; these came up mostly during the consent process. During the consent process, parents learned in greater depth the possible adverse consequences of EIMC. A father told how he had silently posed rhetorical questions during the consent process.

*I kept asking myself, “Isn’t it going to cause some deformities on the thing [penis]?” and “What happens if it [penis] starts decaying?” “What have been the results with regards to this exercise since they are saying it is research?” You know, research normally is research*. (father, in-depth interview)

Parents' initial anxieties came up mostly during the consent process.

Despite assurances that the rate of EIMC-related adverse events is very low and also that, if an adverse event occurs, it is usually minor and easily resolved, most parents remained agitated during the procedure.

*I was so uncomfortable during the time I was sitting here [in the clinic boardroom] as I had been told that my child could possibly get injured during the process*. (mother, FGD)

### Parental Perceptions of Procedure and Outcome

Most parents who had adopted EIMC and who were interviewed after the infant had completely healed thought that the procedure was very safe. A mother remarked:

*Ha-a, it’s safe, 100%; it’s 100% safe. My son healed well. I didn’t have a problem with it. In terms of bleeding, he didn’t bleed that much. It was just some minimal bleeding and that was it. Also, I simply applied Vaseline and he healed quickly. I can say it’s 100% safe*. (mother, in-depth interview)

Another mother expressed similar sentiments:

*It’s safe, the baby did not bleed, he was not sutured, and I did not have to immerse the wound in salt [salty water]*. (mother, in-depth interview)

However, a father’s response suggested some uncertainty:

*Ya-a, it’s safe, but you always wonder if everything is okay because you don’t know and there is no way to know, you understand what I am saying? … You say to yourself, “Let’s hope I don’t end up regretting.” You always have that fear … and you say, “What if something didn’t go well?”… Like I am saying right now, there is no way one can tell whether or not everything was done successfully*. (father, in-depth interview)

Despite assurances from study staff that the procedure had gone well, this participant still had fears that his son’s reproductive capability may have been damaged during EIMC, and he was anxious about his son’s later fertility or sexual functioning. He also felt that he would only be able to tell once his son had grown up and became sexually active. This shows the extent to which some parents view EIMC as a procedure that involves more than just foreskin removal.

Overall, parents who had adopted EIMC expressed satisfaction with the outcome. A mother remarked:

*First of all, it’s smart. Secondly, it [penis] now has a better “shape”… I think that’s why they have the slogan “PINDA MUSMART” [local slogan that promotes VMMC as hygienic]. It’s just a good thing*. (mother, in-depth interview)

On the whole, parents who had adopted EIMC said they would circumcise their next newborn son. Furthermore, they stated that they would recommend EIMC to other parents.

Parents who had adopted EIMC would recommend EIMC to other parents.

### Parents’ Reasons for Not Adopting EIMC

#### Fear of Harm

Fear of immediate harm emerged as one of the major reasons that parents did not adopt EIMC. A few parents expressed concerns that the procedure and its associated complications (e.g., excessive bleeding) could possibly lead to an infant’s death. A father maintained:

*Ya-a, something might be said to be safe, but at the same time, everything has loopholes.… Somehow it might not succeed, you understand? It’s just a simple operation you know. An operation on the appendix is supposed to be safe. A lot of people have undergone that kind of operation, and some have died. There are things that we presume to be safe but are not necessarily safe*. (father, in-depth interview)

Such a concern as this one was based on the assumption that the infant’s penis was not only tiny but also “too fragile” for the procedure. A father asked:

*How do you really know that the child’s foreskin starts here and ends there? What if they overdo it and end up cutting some veins … ?* (father, FGD)

Although some men mentioned that they had decided on EIMC for their sons because they themselves had undergone VMMC, it appeared that if fathers had found VMMC very painful, they were less likely to agree with male circumcision for their sons.

*What you as an adult go through … you imagine the pain and your son undergoing such pain at that age …* (father, FGD)

Another father noted:

*… It [pain] was something that I still remembered very well … I thought of the pain that I had gone through and I refused …* (father, FGD)

During phone interviews, a few parents also mentioned fear of pain as one reason for ultimately deciding against EIMC (Table).

**TABLE:** Telephone Interview Responses: Parental Reasons for Defaulting on Early Infant Male Circumcision

Theme	Subtheme	No. of Responses
Parent refused	**Baby’s father refused**	9
	• *The father refused*. (8 responses) • *I really want but my husband does not want to hear anything about it*. (1 response)	
	**Baby’s mother refused**	5
	• *His mother refused*. (4 responses) • *My wife is refusing to bring him to the clinic*. (1 response)	
Someone else refused	**Wider family refused**	4
	• *My mother-in-law refused*. (2 responses) • *My in-laws are against the idea*. (2 responses)	
**SUBTOTAL**		**18**
Son still too young	**Too young to undergo procedure**	12
	• *He is still too young*. (9 responses) • *It is still too early to circumcise him*. (2 responses)*• It is too early; he is only 6 days old*. (1 response)	
	**Too young to be taken out in public**	4
	• *My baby is too young to be moved around*. (2 responses) • *My baby is too young to mingle with the public*. (2 responses)	
**SUBTOTAL**		**16**
Fear of harm	**Fear of immediate harm**	11
	• *It is too painful*. (4 responses) • *The mother is afraid it may not go well*. (1 response) • *It’s my first child. What if it doesn’t go well?* (1 response) • *I do not want any sleepless nights*. (1 response) • *My wife fears that the wound will take long to heal*. (1 response) • *I am worried about what will happen to the removed foreskin*. (1 response) • *The time for him to get circumcised has already passed*. (1 response) • *It will ruin my marriage*. (1 response)	
	**Fear of future harm**	3
	• *His peers will laugh at him when he is grown up*. (2 responses) • *It might create problems for him in future*. (1 response)	
**SUBTOTAL**		**14**
Son to be circumcised later	**Near future**	8
	• *I will come after his umbilical stump has fallen off*. (3 responses) • *I will bring him after 6 weeks*. (2 responses) • *I will come later; I first need to heal myself*. (2 responses) • *I will come after the pain from injections subsides*. (1 response)	
	**Later**	3
	• *Will come when the baby is older*. (2 responses) • *We will consider it when he turns 5*. (1 response)	
**SUBTOTAL**		**11**
Held up by decision making	**Decision making in progress**	6
	• *We need more time to think about it*. (2 responses) • *I am still thinking about it*. (1 response) • *As a mother, I cannot decide*. (1 response) • *My wife and I have not thought about it*. (1 response) • *I am still trying to convince my husband*. (1 response)	
	**Still awaiting someone’s approval**	4
	• *The father is not around so I cannot decide*. (1 response) • *I am still waiting for the father to give the go-ahead*. (1 response) • *I am still waiting for his grandmother’s approval*. (1 response) • *I still need to hear from my in-laws*. (1 response)	
**SUBTOTAL**		**10**
Male circumcision not part of family tradition	**Male circumcision not practiced in clan**	4
	• *No one has ever done that in our tribe*. (2 responses) • *It is not part of my culture*. (2 responses)	
	**Father not circumcised**	3
	• *I am not circumcised myself*. (2 responses) • *He has to be like his father, who is not circumcised*. (1 response)	
	**Older brothers not circumcised**	3
	• *His brothers are not circumcised*. (3 responses)	
**SUBTOTAL**		**10**
Son to decide for himself	**Do not want to decide for him**	6
	• *He will decide for himself when he grows up*. (6 responses)	
	
**SUBTOTAL**		**6**
External influence	**Wider family’s influence**	2
	• *My older sister advised me not to*. (1 response) • *My mother-in-law discouraged me from doing so*. (1 response)	
	**Health care workers’ influence**	2
	• *My doctor said I should not do it*. (1 response) • *My aunt who is a nurse said we should not just accept new things*. (1 response)	
**SUBTOTAL**		**4**
Son to be circumcised at home	**Grandmother to perform procedure**	3
	• *His grandmother will circumcise him*. (3 responses)	
**SUBTOTAL**		**3**
Other	**Various other reasons**	3
	• *Baby died*. (1 response) • *We are no longer in Harare*. (1 response) • *I will only do it if it is for medical reasons*. (1 response)	
**SUBTOTAL**		**3**
**TOTAL**		**95**

#### Cultural Beliefs

Discussions suggested that cultural beliefs were a significant barrier to EIMC. For example, participants suspected that Satanists were conducting the EIMC research. A mother professed her initial fears:

*I don’t want to lie; I suspected that you were Satanists and that you may take the foreskin … I really don’t know the process, but I was just scared because I don’t want to lie. Satanists are present but we don’t know the organizations they work for …* (mother, in-depth interview)

Another female participant who had adopted EIMC described how someone had scared her after the procedure.

*I met a certain woman who asked me if I had seen where the foreskin had gone. When I told her that I had not seen it but I had only been told that it would be incinerated, she said it was a real shame that I had chosen to sacrifice my child to Satanism. She insisted that I was supposed to have requested the foreskin and disposed of it myself*. (mother, in-depth interview)

Men who did not choose EIMC for their sons stated in a group discussion that they would circumcise their sons only on condition that they would be allowed to take the removed foreskin with them.

#### Advice From Health Care Workers

An additional reason for non-adoption of EIMC was advice from someone considered an expert. In a phone interview, one respondent said, *“My doctor said I should not do it”* (Table). Another maintained, *“My aunt, who is a nurse, said we should not just accept new things”* (Table). Health care workers with inadequate knowledge of the procedure thus played a role in discouraging parents from adopting EIMC.

Health care workers with inadequate knowledge of the procedure played a role in discouraging parents.

### Non-Study Health Workers’ Misconceptions

Discussions suggested that despite several EIMC sensitization meetings with non-EIMC clinic staff, including an initial stakeholder meeting convened by the Ministry of Health, misconceptions remained prevalent among some of these health care workers.

*We understand that the babies are circumcised “just like that,” under no anesthesia. Most nurses therefore think that this program is cruel*. (non-EIMC health care worker, in-depth interview)

Perceptions such as these were a result of poor knowledge of the EIMC procedure; in fact, local anesthetic was applied prior to the procedure.

Misconceptions were also prevalent among ancillary staff, including lay community health workers. The lay community health workers participating in the study were mostly hand-picked elderly women, many of whom had some basic education. A nurse said about them:

*They [lay community health workers] think the infant has to be anesthetized first [general anesthesia], so they assume that if infants are anesthetized, they will “never wake up” [implying dying]. Some have heard of people who have died after being anesthetized*. (non-EIMC health care worker, in-depth interview)

The same nurse went on to state that these community workers often passed on misinformation to the communities where they worked. Given their misconceptions about EIMC, perhaps it is not surprising that the 50 lay community health workers based at the study clinic (and who had been trained by Ministry of Health and study staff) did not refer a single infant for EIMC during the comparative trial, despite initial assurances that they would actively support the study.

Discussions with non-EIMC study nurses also suggested that some of their peers gave mothers ambivalent or contradictory advice regarding EIMC.

*You hear them saying, “Ya-a, it’s a good thing to have him [son] circumcised, but if it was my son, I would not have him circumcised.”* (non-EIMC health care worker, in-depth interview)

This quotation suggests once again that some health care workers did not promote EIMC.

### Study Clinicians’ Perceptions: EIMC Procedure, Devices, and Outcome

During discussions, study clinicians reported their initial anxieties with EIMC. A study doctor described her initial feelings:

*Before I underwent training, I thought EIMC was such a difficult thing. I couldn’t imagine infants being circumcised; I had never seen such a thing.… And then during the lectures when I was taught the various techniques and their complications, it was a bit scary*. (EIMC health care worker, in-depth interview)

Another doctor described the EIMC experience as “mind-opening.” He elaborated:

*I had a preconception that the neonatal stage was the child’s most delicate period and that EIMC could predispose them to infections, would result in delayed wound healing, or anything like that. I never thought circumcision could be that safe and easy*. (EIMC health care worker, in-depth interview)

Despite acknowledging initial anxieties, study doctors stated that, with more exposure and practice, they became confident conducting EIMC and subsequently began to feel that the procedure was both uncomplicated and safe.

*Now I am very comfortable. I now feel that EIMC is a procedure that you can even do with your eyes closed*. (EIMC health care worker, in-depth interview)

Another doctor maintained:

*I think it’s a procedure that can safely be done not only by doctors but by any adequately trained health care provider*. (EIMC health care worker, in-depth interview)

Study nurse-midwives also stated that, after assisting doctors with EIMC procedures, they now felt that they could safely perform the procedure.

*From what I have observed, I am very confident that I can also perform the procedure*. (EIMC health care worker, in-depth interview)

When asked if she thought that nurses other than those involved in the EIMC study could also perform the procedure, a study nurse-midwife responded:

*They can. They are doing episiotomy; episiotomy is cutting a part that is even unmarked, a section you think the unborn baby might come through*. (EIMC health care worker, in-depth interview)

#### Preference for AccuCirc

Discussions suggested that on the whole, study doctors preferred the AccuCirc device over the Mogen clamp due to the former's safety features. A study doctor said:

*AccuCirc is straightforward and it’s a device that can protect. … We have that thing that protects the glans, so you don’t have to worry about anything …* (EIMC health care worker, in-depth interview)

Another study doctor explained why he preferred the AccuCirc device over the Mogen clamp:

*I have realized that when using AccuCirc, there is less manipulation of the penis. With Mogen clamp, you manipulate the foreskin so much that the pen mark disappears just before you cut off the foreskin and so you won’t have a good approximation of the amount of foreskin that you should remove. As a result, you can either remove too little or too much foreskin*. (EIMC health care worker, in-depth interview)

#### Clinicians Like Outcome

Just as with parents who adopted EIMC, study clinicians stated that the EIMC outcome was aesthetically pleasing. A study nurse-midwife commented:

*It’s indeed a sweet outcome; you actually feel proud of it*. (EIMC health care worker, in-depth interview)

A study doctor felt that, compared with adult male circumcision, EIMC produced a better cosmetic result.

*EIMC has amazing results even when compared to adult MC [male circumcision]. I most liked the fact that the outcome was not only satisfactory to us as providers but to the parents and guardians of the infants as well*. (EIMC health care worker, in-depth interview)

### Clinicians’ Perceptions: Feasibility of Wide-Scale EIMC

Informed by their experience with EIMC in the context of a small research trial, health care workers felt that it was feasible to roll out wide-scale EIMC for HIV prevention in Zimbabwe. On the whole, these health care workers felt that AccuCirc was likely to have several advantages over the Mogen clamp when it came to wide-scale implementation, including, for example, through rural clinics.

*Even nurses stationed at rural areas will be able to safely perform the procedure through AccuCirc, especially because the device is simple to use and the shielding ring makes it impossible to partially amputate the penile glans*. (EIMC health care worker, in-depth interview)

Non-study nurses expressed optimism that, if adequately trained, they could safely perform EIMC using the AccuCirc device.

*The other time one of the study guys had a meeting with us, he clearly demonstrated how that white plastic gadget [AccuCirc] works. If we are trained, we will be able to perform the procedure. Also, we already have knowledge of some of the aspects such as sterile procedures. We can do it*. (non-EIMC health care worker, in-depth interview)

Despite overwhelmingly recommending that nurses and midwives be trained and delegated to perform EIMC, nurses (both EIMC and non-EIMC) felt that these clinicians should be covered by doctors in case of any major complications.

### Clinicians’ Concerns

#### Dealing With Parental Anxieties

During the EIMC comparative trial, some parents called study staff whose mobile numbers were on the contact card (including at night) to report issues that the staff thought trivial (e.g., that the baby had soaked the bandage with urine). EIMC study clinicians wondered how these understandable parental anxieties would be addressed in the context of roll-out. Specifically, they questioned how the public health sector would be able to provide such intensive phone support.

*Will the public clinics and hospitals be able to put in place the necessary supportive mechanisms such as taking night phone calls?* (EIMC health care worker, in-depth interview).

#### Wound Care Management

EIMC study clinicians stated that, unlike disposable diapers, cloth diapers (the option used by most people) are less absorbent and perhaps changed less frequently, so that the baby was sitting in damp (cloth) diapers for long periods, resulting in delayed wound healing.

*… Some delayed wound healing we experienced had to do with the fact that mothers were using nappies [cotton or cloth diapers], but once we gave them the Pampers [disposable diapers], the babies subsequently healed very well*. (EIMC health care worker, in-depth interview)

The same doctor went on to question the feasibility of purchasing large volumes of disposable diapers during EIMC roll-out.

*Will you be able to purchase Pampers [disposable diapers] for all those babies whose parents cannot afford Pampers during the roll-out?* (EIMC health care worker, in-depth interview)

## DISCUSSION

We nested a qualitative study within a trial that assessed the feasibility, safety, acceptability, and cost of rolling out EIMC using devices in Zimbabwe. The qualitative study enabled us to explore parental and health care workers' perspectives on EIMC conducted using devices, especially as they relate to EIMC safety, acceptability, barriers, and feasibility.

Despite acknowledging initial anxieties with EIMC, parents expressed satisfaction with both the procedure and the outcome. These findings corroborate the EIMC trial findings, in which nearly all mothers (99.5%) reported satisfaction with the outcome,^16^ and are consistent with findings from other regional settings, which also have found high levels of satisfaction with the EIMC outcome (Botswana >94%, Kenya 96%, and Zambia 96%).^18^^,^^19^^,^^26^ Once EIMC is rolled out, perceptions of the safety and aesthetic aspects of EIMC will have a bearing on whether or not uptake of the procedure will be sustained.^27^ To maintain high levels of satisfaction within EIMC programs, provision of the procedure will need to be carefully supervised and monitored to ensure (1) a good cosmetic result and (2) that adverse events are prevented.^14^

Despite initial misgivings, both parents and the clinicians who performed the circumcisions expressed satisfaction with the procedure and its outcome.

Like parents, study clinicians, despite acknowledging initial anxieties with EIMC, reported satisfaction with EIMC. All felt that it is a simple procedure that can be performed by non-doctors. These opinions are especially important because they were expressed by health care workers experienced with the procedure. Moreover, based on their experience, clinicians felt that it was feasible to offer wide-scale EIMC for HIV prevention. Their recommendations need to be considered when planning EIMC scale-up, especially their recommendations that nurse-midwife providers should be covered by doctors in case of any major complications and that the AccuCirc device should be used during roll-out. Furthermore, issues concerning dealing with parental anxieties and EIMC wound management need to be carefully considered in the context of scale-up. The study also found a poor understanding of EIMC among health care workers not involved in conducting the procedure, highlighting the importance of adequate training of these stakeholders.

Parental reasons for non-adoption of EIMC included fear of harm, cultural beliefs, and misinformation from health care workers. These findings are similar to those of EIMC hypothetical acceptability studies.^14^^,^^28^^–^^32^ These recurring barriers highlight once again the need to enhance EIMC knowledge among both parents and health care workers. Information, education, and communication materials will need to provide understandable and accurate information explaining the procedure and state clearly that, when conducted by appropriately trained and experienced personnel, EIMC is safe.^16^^,^^18^^,^^19^

Additionally, communication materials need to highlight the several advantages of circumcising males during infancy rather than later in life. The materials also will need to include a “frequently asked questions” section that specifically addresses the parental concerns raised in this and other studies. Engaging satisfied parents to promote circumcision will be an important strategy to allay other parents’ fears. It may also be important to develop quality standards to assess provider–client communication, with a view to assuring the quality of the EIMC counseling process.

Engaging satisfied parents to promote circumcision will be an important strategy to allay other parents’ fears.

The finding that painful VMMC experiences among adult men leads them to reject EIMC for their sons has at least 3 implications for VMMC roll-out in general and EIMC promotion in particular. First, adult VMMC programs need to manage clients’ pain adequately. Second, VMMC clients circumcised via conventional surgery need to be empowered so that during the procedure they will alert providers whenever the anesthesia is not adequate. Last, EIMC awareness campaigns need to explain the differences between adult conventional surgery and EIMC—especially the fact that the latter does not require an anesthetic injection, sutures, or dipping the post-circumcision wound in salty water, often considered painful.^33^

Correcting misconceptions around VMMC in general and EIMC specifically will be another important strategy in awareness campaigns to allay parental concerns. Awareness campaigns need to specifically address the persistent concern about the discarded foreskin.^14^^,^^31^ The wider community needs to be informed that all removed foreskins will be incinerated according to national and international tissue disposal policies. It will be difficult for parents to obtain the discarded foreskin from EIMC clinics since it is likely that EIMC will be rolled out using the AccuCirc device, which is designed to retain the discarded foreskin; parents would need to obtain the entire used AccuCirc device in order to get the foreskin. This in turn would have implications for waste disposal, as AccuCirc is made largely of hardened plastic and will take years to biodegrade. A possible strategy would be to invite parents to see how the device is disposed of and to have community leaders witness the incineration process as a way of enhancing communities' trust in the disposal process.

Awareness campaigns need to specifically address the persistent concern about the discarded foreskin.

### Strengths and Limitations of the Study

This qualitative study was nested within the first randomized comparison of AccuCirc and Mogen clamp devices for EIMC in sub-Saharan Africa. The advantages of conducting qualitative research with or within trials are now well recognized. For one thing, the qualitative components of trials gather information that helps to answer research questions in depth.^34^^,^^35^ In this case, the qualitative research brought out parents’ reasons for non-adoption of EIMC and thus helped to explain the low rate of EIMC uptake during the trial.

This study also triangulated various data collection methods (focus group discussions, in-depth interviews, and short phone interviews) to explore the *actual* as opposed to *hypothetical* acceptability and feasibility of EIMC among parents, study clinicians, and non-study clinicians. Triangulation has been widely adopted in qualitative research as a means to investigate the validity of both the data and the conclusions derived from them.^24^ In this case, there was concordance among data obtained through focus group discussions, in-depth interviews, and short phone interviews, reinforcing the likely validity of the results. Of note, many of the issues raised here also arose in qualitative studies designed to investigate hypothetical acceptability.

A potential limitation of this study is that budget and time constraints limited our sample to 12 in-depth interviews, 4 focus group discussions, and 95 short phone interviews with parents. It could be argued that parents with newborn sons who declined to participate in the trial (n = 984) were not adequately represented. It is also possible that we did not fully explore all themes due to the relatively small sample.

## CONCLUSIONS

This qualitative study enabled us to explore parental and health care workers’ perspectives on EIMC conducted using devices. Parents who opted for EIMC and EIMC providers felt that EIMC was a safe and acceptable procedure that would likely be more widely adopted over time. However, some parents and health care workers without experience or knowledge of EIMC feared that the procedure might be harmful or painful. These findings will be used to inform the design of a demand-generation intervention to support wider adoption of EIMC.

Parents who opted for EIMC and EIMC providers felt that EIMC was a safe and acceptable procedure that would likely be more widely adopted over time.

## Notes

**Peer Reviewed**
